# Molecular Dynamics Simulations Enforcing Nonperiodic Boundary Conditions: New Developments and Application to the Solvent Shifts of Nitroxide Magnetic Parameters

**DOI:** 10.1021/acs.jctc.2c00046

**Published:** 2022-03-08

**Authors:** Giordano Mancini, Marco Fusè, Filippo Lipparini, Michele Nottoli, Giovanni Scalmani, Vincenzo Barone

**Affiliations:** † Scuola Normale Superiore, Piazza dei Cavalieri 7, 56126 Pisa, Italy; ‡ Istituto Nazionale di Fisica Nucleare (INFN) sezione di Pisa, Largo Bruno Pontecorvo 3, 56127 Pisa, Italy; ¶ Dipartimento di Chimica e Chimica Industriale, Universitaá di Pisa, Via G. Moruzzi 13, 56124 Pisa, Italy; § Gaussian, Inc., 340 Quinnipiac Street, Building 40, Wallingford, Connecticut 06492, United States

## Abstract

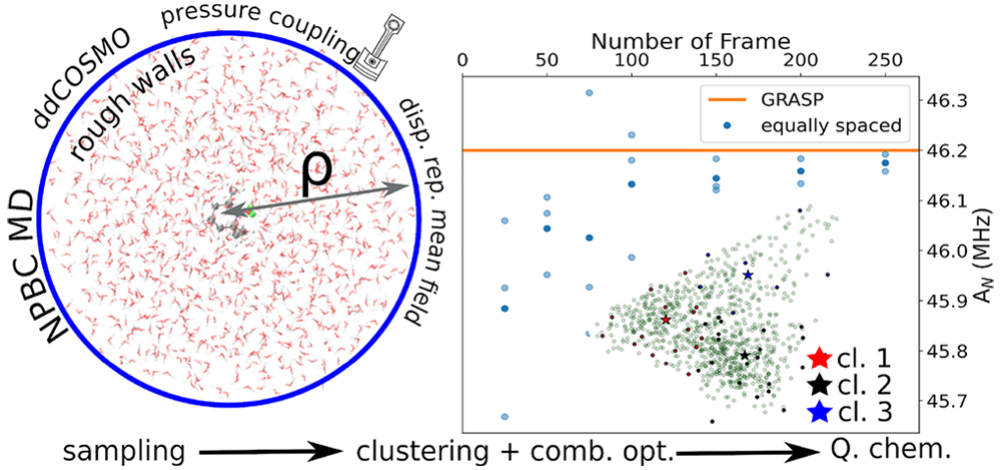

Multiscale methods combining quantum mechanics and molecular mechanics (QM/MM) have become the most suitable and effective strategies for the investigation of the spectroscopic properties of medium-to-large size chromophores in condensed phases. In this context, we are developing a novel workflow aimed at improving the generality, reliability, and ease of use of the available computational tools. In this paper, we report our latest developments with specific reference to a general protocol based on atomistic simulations, carried out under nonperiodic boundary conditions (NPBC). In particular, we add to our in house MD engine a new efficient treatment of mean field electrostatic contributions to energy and forces, together with the capability of performing the simulations either in the canonical (*NVT*) or in the isothermal–isobaric (*NPT*) ensemble. Next, we provide convincing evidence that the NBPC approach enhanced by specific tweaks for rigid body propagation, allows for the simulation of solute–solvent systems with a minimum number of degrees of freedom and large integration time step. After its validation, this new approach is applied to the challenging case of solvatochromic effects on the electron paramagnetic resonance (EPR) spectrum of a prototypical nitroxide radical. To this end, we propose and validate also an automated protocol to extract and weight simulation snapshots, making use of a continuous description of the strength of solute–solvent hydrogen bridges. While further developments are being worked on, the effectiveness of our approach, even in its present form, is demonstrated by the accuracy of the results obtained through an unsupervised approach characterized by a strongly reduced computational cost as compared to that of conventional QM/MM models, without any appreciable deterioration of accuracy.

## Introduction

A general, robust, and effective strategy for the computation of spectroscopic parameters in solution can be broken down in a number of successive steps. The first step is the generation of a sufficient number of representative structures by means of atomistic simulations, such as Monte Carlo (MC) or Molecular Dynamics (MD). Next, suitable clustering procedures can be employed to reduce the number of different structures needed to obtain a statistically significant ensemble, able to provide well converged average results. Finally, reliable yet effective multiscale methods combining quantum mechanics and molecular mechanics (QM/MM) can be employed to compute averaged values of the desired properties.^1^ While in principle all steps can be performed at the same time by extended Lagrangian^2,3^ or Born–Oppenheimer^4,5^*ab initio* molecular dynamics, both the space and time scales which can be covered at present by these approaches are too small to deliver converged results for medium-to-large size chromophores. Furthermore, the accuracy required in the computation of the spectroscopic parameters is usually much higher than that needed in the generation of the representative snapshots. Alternatively, quite simple molecular mechanics (MM) force fields are now available for describing solvent dynamics and also solute–solvent interactions can be usually modeled quite effectively by well-tested force fields. The situation is however different for the computation of spectroscopic parameters, where a quantum mechanical description of the solute (and possibly its cybotactic region) is unavoidable and solvent polarization can have a non-negligible effect.^6^ A companion paper deals with an effective strategy for this last step, exploiting a QM/MM polarizable model based on the floating charge (FQ) approach,^7^ including effective recipes for taking into account solvent–solvent charge-transfer,^8^ and correcting the wrong coordination numbers of solvent molecules near the outer boundary of the simulation box.^9^ Here, we will focus on the initial atomistic simulation and the clustering of the resulting trajectories in the framework of our computational engine for classical molecular dynamics simulations employing a spherical simulation box and nonperiodic boundary conditions (NPBC).^10,11^ In particular, we will describe a new quasi-analytical implementation of reaction field effects at the outer boundary of the simulation sphere by means of a series expansion of the polarization density, in the spirit of the domain decomposition conductor-like screening model (ddCOSMO),^12,13^ for MD simulations employing either the canonical (*NVT*) or the isothermal–isobaric (*NPT*) ensemble. In the latter case, a new thermostat and an effective treatment of the simulation sphere breathing have been introduced. After parametrization and simulation of an effective water model (TIP3P-FB^14^), the overall computational strategy has been applied to a quite demanding case study, namely the tuning of the magnetic properties of a representative nitroxide radical (TEMPO, i.e., (2,2,6,6-tetramethylpiperidin-1-yl)oxidanyl) by different solvents.^15^ To this end, we have performed a preliminary benchmark study for the model dimethyl nitroxide radical, taking as references the results obtained by state-of-the-art QM calculations performed specifically for this system. After validating the QM model, the new NPBC molecular dynamics engine is used to generate a statistically significant number of snapshots to take into account solvent modulation effects. Extending the pipeline commonly used to select significant structures or subtrajectories from MD simulations,^16−18^ we adopt a two-step procedure: First, we define distinct basins using a clustering^19^ procedure in which optimal clusters are found by means of an optimization of validity scores. Next, following a strategy reminiscent of that employed with success for electronic spectra,^11^ a representative structure for each basin is treated at the QM/MM level, including in the QM subsystem a few solvent molecules, whereas fluctuations within each basin are taken into account by computing a sufficient number of structures with a cheaper approach in which the QM part is reduced to just the solute. The whole procedure can be cast in terms of a fully unsupervised workflow, so that, together with the intrinsic interest of the specific system, the validation of such protocol paves the route toward the study of several other problems of current technological or biological interest.

## Methods

### NPBC Simulations

The framework of our computational strategy is the GLOB model^10^ in which a spherical cavity with rigid boundary contains the proper number of solvent molecules needed to enforce an average density equal to the experimental counterpart. Optionally, a solute can be also placed at the center of the cavity, removing the solvent molecules within van der Waals distances from any of its atoms. Next, two soft potentials are added in order to describe the reaction field of the solvent outside the cavity (*U*_*RF*_) and to enforce a nearly constant density in the whole simulation sphere (*U*_*vdW*_).

### Reaction Field for a Spherical Cavity

In this section, the main equations that describe the reaction field contribution for a spherical cavity are presented, and their implementation is discussed in some detail. Let Ω be a spherical cavity of radius *R* centered at *C*_0_ and Γ = ∂Ω its boundary. Given an electrostatic distribution ρ fully supported inside Ω, we want to solve the Conductor Polarizable Continuum Model^20^ (C-PCM) or, equivalently, of the Conductor-like Screening Model^21^ (COSMO) electrostatic equation
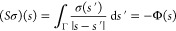
1where σ is an apparent surface charge (ASC) supported on Γ and Φ is the molecular electrostatic potential generated by the solute’s charge distribution (q_*k*_’s at positions r_*k*_’s)
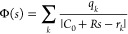
2Given the spherical symmetry of the problem, it is convenient to expand both the potential and the ASC in spherical harmonics, following the procedure introduced in the ddCOSMO algorithm.
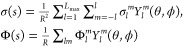
3There we introduced a compact notation for the *l*, *m* sum and *L*_max_ is the angular momentum to which we truncate the expansion. Note that, with respect to previous publications of the ddCOSMO method, we have introduced a slightly different scaling of the potential and ASC with respect to the cavity radius.

Using the spherical harmonics addition theorem, the COSMO integral equation becomes
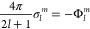
4

The solvation energy can then be computed as
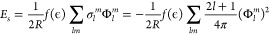
5The spherical harmonics expansion coefficients of the potential needed to solve eq 5 can be easily computed using a quadrature, with the Lebedev–Laikov (LL) ansatz being particularly well suited due to the symmetry of the problem. Let *w*_*n*_, *s*_*n*_ be the LL weights and points:
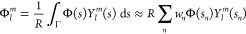
6

The contributions to the forces are obtained by straightforward differentiation of eq 5. Let
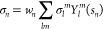
7We get, on atom *k*,
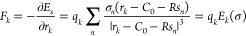
8where we can recognize the electric field produced by σ_*n*_ distribution at the *k*th site.

For simulations at constant pressure, the derivative of the solvation energy with respect to the volume is needed. The latter can also be easily computed by differentiating eq 5 with respect to *V* = 4/3*πR*^3^:
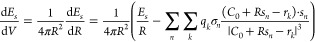
9where we can rewrite the last term by recognizing the electric field produced by the solute’s charge distribution at the cavity:
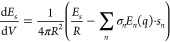
10

From a computational point of view, the only quadratic scaling terms in all the equations correspond to the evaluation of the electrostatic potential or field of a charge distribution, which can be performed at a cost that scales linearly with the number of atoms by using a fast summation technique. In our implementation, in particular, we use the Fast Multipole Method.^22,23^ The evaluation of Spherical Harmonics at the Lebedev points, which is also required in various parts of the computation flow, is achieved as extensively documented in ref (24). For the sake of brevity, in the following, we will refer to the electrostatic solvation model simply as ddCOSMO, as it indeed corresponds to a ddCOSMO treatment of a single spherical cavity.

### Isothermal Isobaric Ensemble and Weak Coupling

In this study, we have adopted the simplest available pressure control algorithm, namely the so-called Berendsen barostat^25^ with the purpose of assessing the stability of the MD trajectory in conjunction with the ddCOSMO model and a breathing cavity. The pressure tensor **P** can be calculated using Clausius’ virial theorem as^25^
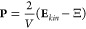
11where *V* is the box volume, **E**_*kin*_ is the kinetic energy, and Ξ is the virial tensor:
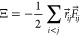
12with  and  being the coordinates and forces, respectively. In the framework of spherical NPBC, the isotropic pressure is
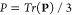
13Since in a *NPT* simulation *E*_*kin*_ is coupled to the thermostat, the pressure can be controlled by changing the virial, i.e., by scaling the particle positions and, in the present case, the sphere radius *R*. This is achieved by weakly coupling the system to an external piston, i.e., by letting the pressure relax according to
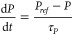
14where τ_*P*_ is the time constant for the coupling. The relationship between the pressure and the isothermal compressibility β (incorporating the factors for different box types in the α parameter) is
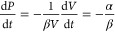
15which can be used to calculate the scaling factor μ needed to scale coordinates and radius from  to  (and from *R* to *μR*) in a single time step *δt*:
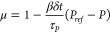
16

### Computational Details

#### MD Simulations

The MD simulations presented in this work include different solvents (CH_3_CN, CH_3_OH, and two different water models, namely SPC^26^ and TIP3P-FB^14^) and, possibly, the TEMPO solute. We choose to use the TIP3P-FB water model for solute–solvent simulations due to its improved bulk properties with respect to the SPC counterpart, including dipole moment (2.43 vs 2.2 D), density (0.995 vs 0.960 g/cm^3^) and static dielectric constant (81.3 vs 65). The topology of the TEMPO solute was adapted from the study of Stendardo et al.,^27^ discarding intramolecular interactions since we kept the molecular structure frozen in all the simulations. In this model, two virtual sites are used to describe the lone-pairs of the nitroxide oxygen. The topology and force field parameters of the nonaqueous solvents were the same used in previous studies,^10^ whereas the details of the different water models are given in the original publications quoted above.

All MD simulations were run with a locally modified version of the Gaussian^28^ suite of programs using the rotational velocity Verlet (RVV1) integrator^29^ with an ϵ = 10^–9^ convergence criterion for the calculation of quaternion derivatives, with the exception of CH_3_OH which was treated as a flexible molecule and simulated employing the standard Velocity-Verlet integrator and enforcing holonomic constraints by means of the RATTLE method.^30^ The Bussi–Donadio–Parrinello^31^ thermostat was used, and a weak coupling scheme was employed in *NPT* simulations. Reaction field effects at the outer boundary of the simulation sphere were taken into account by the ddCOSMO model. Mechanical restraints were imposed instead by “rough walls”, such that whenever a solvent molecule goes outside the simulation sphere, its center of mass (COM) velocity is aligned along a new direction sampled with uniform probability in a unit sphere, with a new magnitude corresponding to the average velocity at the thermostat temperature, and the angular momentum is reset in the same way, generating a new angular velocity.

All the starting structures were initially brought to a low-energy configuration with a conjugate gradient minimization. Runs were started sampling initial COM velocities and angular momenta at 298.15 K and carried out for 5 ns (pure water), 30 ns (mean field optimization), 2.5 ns (*NPT* simulations), and 12.5 ns (TEMPO simulations). The first 1 ns of each trajectory (2.5 ns for TEMPO/solvent) was considered equilibration and was not used in the following analysis.

The complete simulation list includes:

- Pure water with the SPC model (*NVT* with and without ddCOSMO) with increasing box radii: 8.5, 10.5, 12,5, 15.5, 18.0, and 20.0 Å, with the largest box employed also for mean field optimization and TEMPO simulations. The integration time step was 2.0 fs when the radius was 8.5 Å, 4.0 fs otherwise. The thermostat coupling constant was 0.4 ps.
- *NPT* ddCOSMO simulations with two different box radii (15.5 and 20.0 Å), a time step of 2.0 fs, and coupling constants for thermostat and barostat of 0.4 and 4.0 ps, respectively.
- Optimization of a new *U*_*vdW*_ potential for TIP3P-FB water, performing the required MD runs with the same parameters used for the NVT simulations of SPC water.
- Production runs of TEMPO with CH_3_CN, CH_3_OH and TIP3P-FB water solvents employing the same parameters as above with the exception of CH_3_OH (time step of 2.0 ps and thermostat coupling constant of 0.2 ps).

#### Optimization and Fitting of the *U*_*vdW*_ Potential

The optimization of the *U*_*vdW*_ potential to be employed in the GLOB model^32^ proceeds as follows: the simulation sphere is divided in concentric shells and, at a given interval, a tiny repulsive or attractive term localized in the shell is added, depending on past density fluctuations so that at the end these will be damped.^10^ Here we used a total of 80 constant radius shells, updating the density profile every 50000 simulation steps. Other parameters of the GLOB model were set as in previous publications.^10^ When the density fluctuations are small enough the potential energy profile is saved. Since *U*_*vdW*_ acts mainly near the outer boundary of the NPBC sphere, prior to the fitting the profile is truncated once its value is below 0.1 kJ/mol. The potential energy points are split in training and test subsets of the same size, and the profile is finally fitted by a polynomial expression:
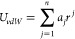
17

The degree of the polynomial is determined running the corresponding ridge regressions. We tested degrees from 0 to 12 and for each degree of the polynomial the shrinking factor value was optimized with a λ, μ Evolutionary Algorithm (EA)^33^ with a population size of 100, mutation rates of 0.3 and 0.5 for parents and children, and a crossover rate of 0.5 for 500 iterations. The degree of the polynomial was finally selected by choosing the best outcome of the corresponding learning curves for the root mean square error (RMSE) and *R*^2^ values.

#### Analysis of Trajectories

The calculation of structural properties was carried out with standard procedures, but taking into account proper normalization for NPBCs. The strength of the hydrogen bonds was calculated with a continuous function^34^ referred to in the following as *F*_*HB*_.^72^

Solvent effects on the solute properties were evaluated for a selection of simulation frames using unsupervised learning methods. We proceeded as follows: first we built a set of coordinates to be used to compare MD frames (the feature space); next, the number of dimensions was reduced by a Principal Component Analysis (PCA) under the constraint of a (projected variance)/(total variance) ratio larger than 0.9. Finally, we used the projected feature space and a clustering procedure to separate the trajectories in different basins, each described by its centroid. Once a centroid was obtained, we sampled from each cluster a number of points proportional to the cluster size in order to obtain a fixed number of simulation frames employing a combinatorial optimization method^35^ to cover both high and low density regions in the projected feature space region of each cluster. The details of each step are described below.

As a feature space for the snapshot selection of TEMPO trajectories we used the first and second neighbor distances for all the N atom1, N atom2, LP_1_-atom1, LP_1_-atom2, LP_2_-atom1, and LP_2_-atom2, where atom1 and atom2 are two properly chosen atoms for each solvent species and LP1, LP2 are the virtual sites of the nitroxide oxygen. We took a frame every 10 ps to build the feature space in order to avoid short time correlations in the clustering. Simulation frames were clusterized by the Partition Around Medoids (PAM) algorithm.^36^ Since cluster analysis without robust validation is meaningless,^17,19,37,38^ the best number of clusters (*k*) was determined from the analysis of PAM runs for values from 2 to 20 in terms of the internal validation criteria of the Silhouette Score (Supporting Information), Dunn Index (DI) and Calinski–Harabasz score (pSF).^36,39^ In addition, we looked for a break-even point in the Within Sum of Squares error (WSS). SI, DI, and pSF should have a maximum corresponding to the parameter set (just the value of *k* in this case) that yields the best clustering while for WSS one looks for a change in the slope. The best value of *k* was obtained from the consensus of three criteria over four.^40,41^

Representative frames within clusters were selected by an in house implementation of the Greedy Randomized Adaptive Search Procedure (GRASP),^42^ which is a procedure to solve combinatorial optimization problems. Given a problem defined by a finite set *E* = {1, 2, ..., *n*} (here the MD frames^43^) and objective function , which measures the total dissimilarity (DS) of the selected snapshots taking into account density, we want to find a set of feasible solutions *F* ⊆ 2^*E*^ (possible subset of frames) which maximizes the objective; i.e., it gives a description as complete as possible of the cluster. Here, we have used an implementation of GRASP made up of two phases,^42^ namely the construction of a feasible solution followed by its refinement employing a local search method. In the first phase, a Restricted Candidate List (RCL) is built adding elements based on their contribution to the total dissimilarity:^44^ a candidate *c* is added if *DS*_*min*_ ≤ *DS*(*c*) ≤ *DS*_*min*_ + α·(*DS*_*max*_ – *DS*_*min*_); i.e., α tunes the algorithm from a greedy to a completely random solution (here we used α = 0.1), and a random element is then chosen from the candidates to build the solution. In the second phase, the built solution is refined selecting elements in the neighborhood of each candidate and checking if the swap gives some improvement. The swap is tried starting from the nearest neighbor and proceeding toward all the points near a specific candidate or for a fixed fraction of the data set (here 0.05). The whole procedure is repeated for a predefined number of times (here 100) selecting the best overall outcome.

## Results and Discussion

### Pure Solvent *NVT* Simulations

#### Boundary Properties of NPBC/ddCOSMO Simulations

**Figure 1:**
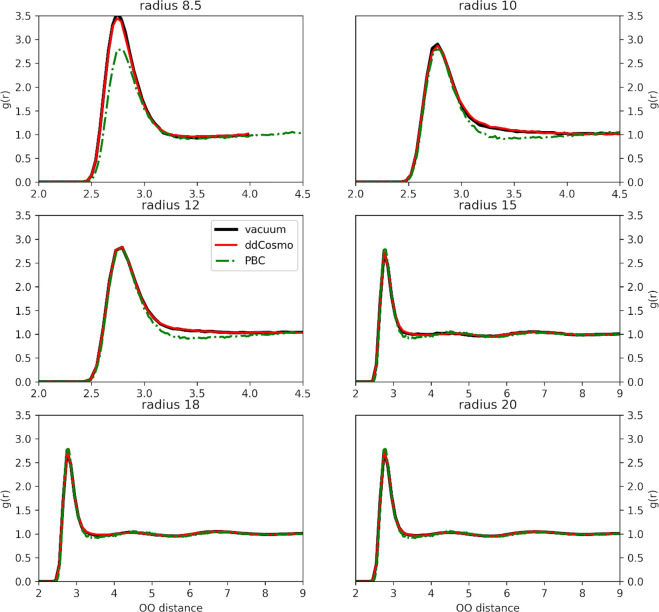
Oxygen–oxygen radial distribution functions of *NVT* pure SPC water simulations under NPBC–vacuum (black solid line), NPBC–ddCOSMO (red solid line), and PBC (green dot dashed line).

Figure 1 shows the OO radial distribution function (*g*(*r*)) obtained from the different pure water simulations. For the smaller simulation spheres (radii from 8.5 to 12 Å) the *g*(*r*) was calculated considering the distribution for all the oxygen atoms within 4.5 Å and looking at neighbor atoms up to 4.0, 5.5, and 7.5 Å, respectively; for the larger systems, we took into account atoms within 8.0 or 10.0 Å and searched for neighbor ones up to 7.0, 8.0, and 10.0 Å, respectively.

**Figure 2:**
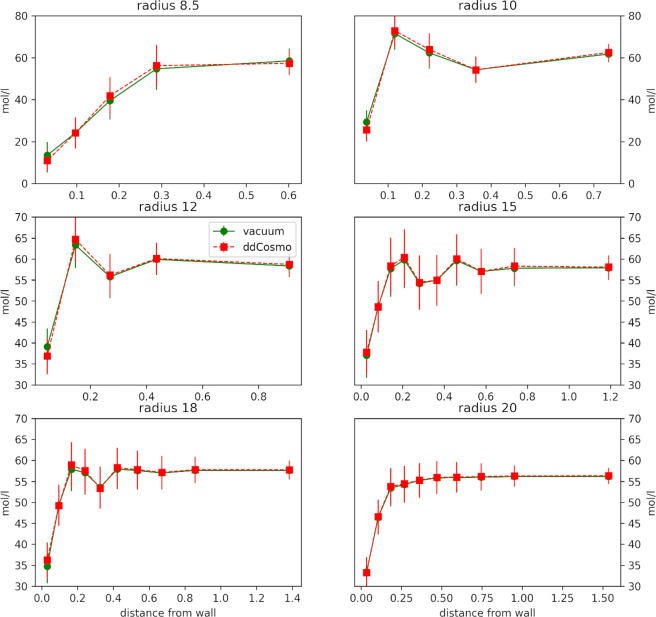
Mean density and standard deviations (error bars) in five (radius 8.5–12 Å) or 10 (radius ≥12 Å) constant volume concentric shells for *NVT* SPC MD simulations.

As can be easily observed, the simulation with the smallest box (8.5 Å radius) not including the reaction field contribution features a first peak of the radial distribution function which is higher and slightly left shifted with respect to the PBC one, indicating a loss of structural disorder. This effect is partially overcome by using ddCOSMO as illustrated by the values of the half weight at half-maximum (HWHM) of 0.188, 0.216, and 0.209 Å calculated at 1.725, 1.518, and 1.385 Å, respectively). This effect already disappears for boxes with radii larger than 10 Å; the second and third plot show a lack of the depletion zone which separates the first and second coordination shell, but even this effect vanishes for radii larger than 15 Å. From a different point of view, the same trend is observed when looking at density fluctuations in concentric shells of constant volume (see Figure 2)

The shortcomings of the SPC model are well-known, and the green curve is not a really good model for the structure of liquid water; however, under the same simulation conditions, the agreement between the liquid structure provided by PBC and NPBC simulations gives a convincing indication of adequate system size and absence of additional artifacts.

### *NPT* Simulations

**Figure 3:**
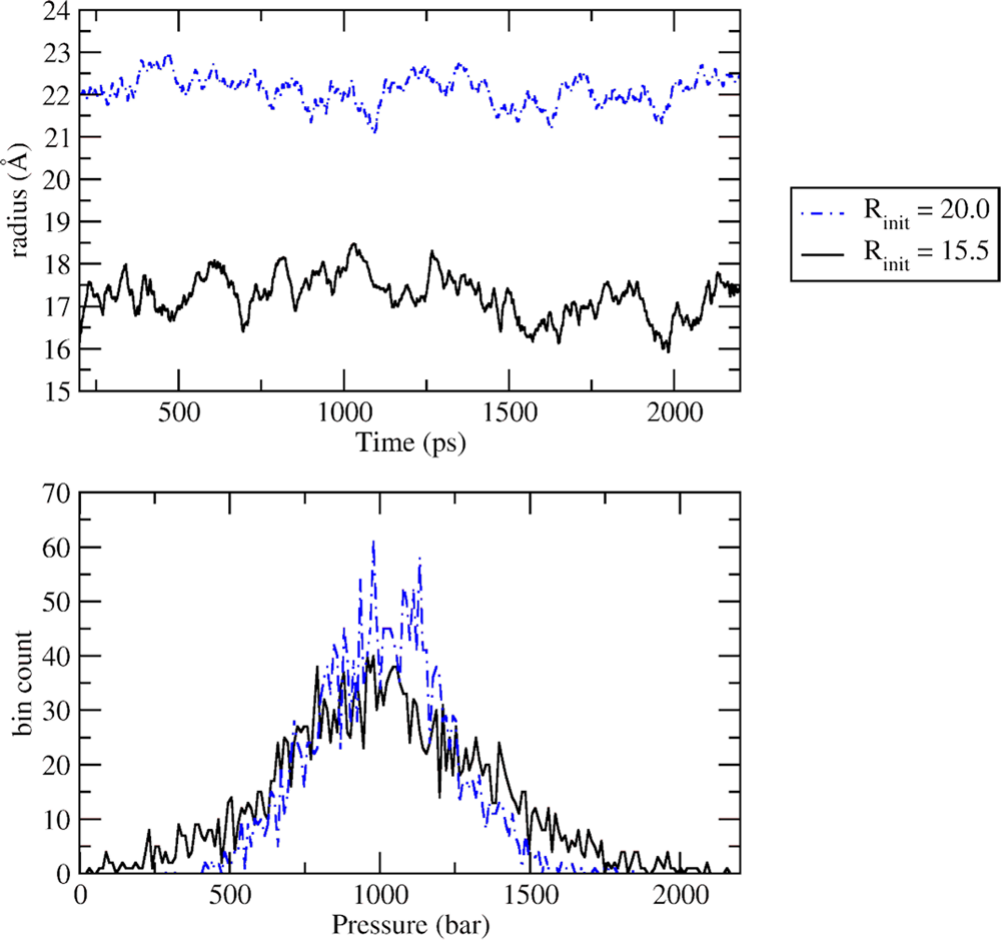
*NPT* simulations, skipping a calibration time of 200 ps. Upper panel: radius of simulations starting at 15.5 and 20.0 Å. Lower panel: histogram of sampled pressure values.

Figure 3 shows the results of both the *NPT* simulations carried out for pure water spherical boxes setting the barostat at 1000 bar.

The starting coordinates and box radii were the same of the corresponding largest *NVT* simulations, i.e., 15.5 and 20.0 Å. The upper panel shows the evolution of the box radius as a function of time after the equilibration time of 200 ps. It is apparent that the average radii of the two simulation spheres (17.15 and 22.10 Å, respectively) are about 2.0 Å larger than the starting values and that they oscillate around the average values. The lower panel shows the histograms of the sampled pressure values where it is apparent that the instantaneous pressure distribution is centered on the barostat value.

In our opinion, these results provide a satisfactory demonstration of the robustness and stability of the approach we implemented.

#### Optimization of the *U*_*vdW*_ Potential for TIP3P-FB Water

Having assessed the stability of the new features for the SPC water model, we ran a 40 ns *NVT* simulation to obtain an optimized *U*_*vdW*_ potential energy profile for TIP3P-FB H_2_O.

**Figure 4:**
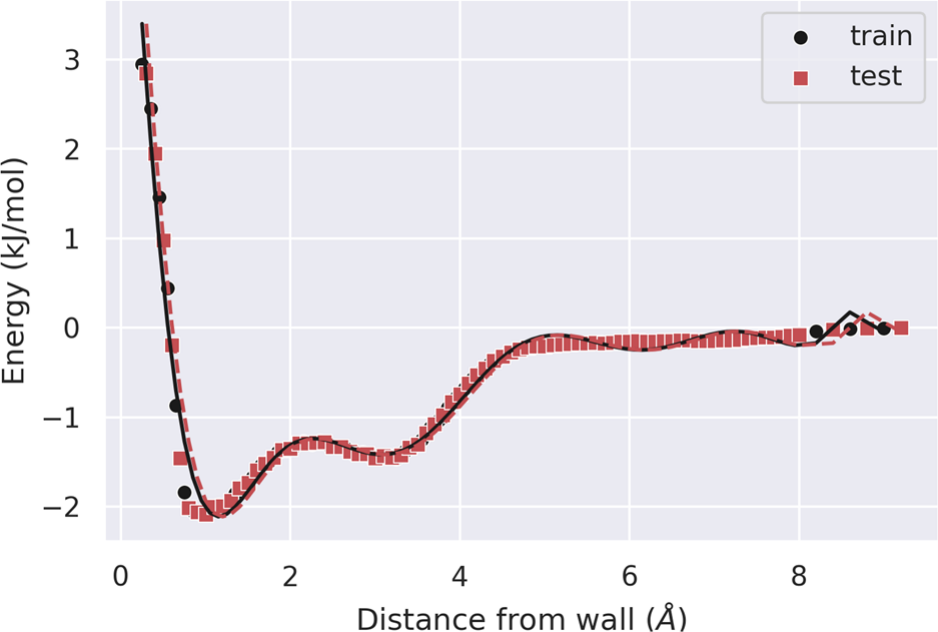
TIP3P-FB mean field potential energy profile. The data used in the fitting and the corresponding fitted polynomial are drawn in black, whereas the test points are drawn in red.

**Figure 5:**
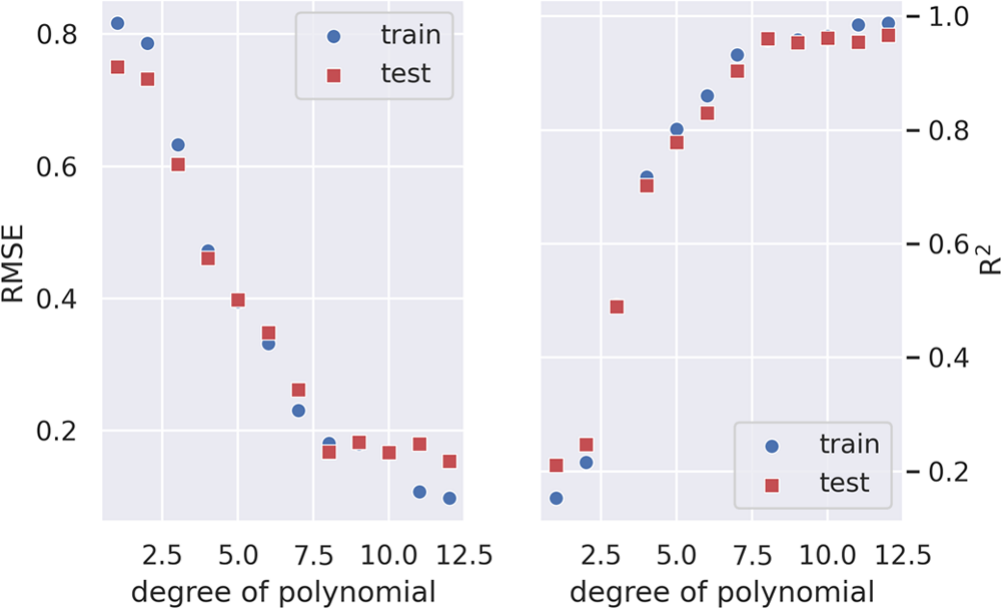
Value of RMSE and *R*^2^ as a function of the degree of the polynomial. Train and test values are superimposed.

The resulting profile is shown in Figure 4 (without the truncated portion) together with the fitted polynomial, whose coefficients are shown in Table 1). Both training and test sets contain 80 points with a resolution of 0.1 Å, after truncation at 10 and 8 Å, respectively. Looking at the learning curves in Figure 5 for RMSE and *R*^2^, we selected a tenth-degree polynomial. Note that the H_2_O profile shows a double well, which explains the higher degree of the fitting polynomial needed for water with respect to organic solvents, as already reported for the SPC water model.^10,32^

**Table 1:** *U*_*vdW*_ Linear Fit Parameters for TIP3P-FB water

parameter	TIP3P-FB
*a*_0_	6.958446e+00
*a*_1_	–1.381768e+01
*a*_2_	–7.095241e+00
*a*_3_	2.678593e+01
*a*_4_	–2.224122e+01
*a*_5_	9.410601e+00
*a*_6_	–2.343013e+00
*a*_7_	3.571387e-01
*a*_8_	–3.280733e-02
*a*_9_	1.669372e-03
*a*_10_	–3.616741e-05

**Figure 6:**
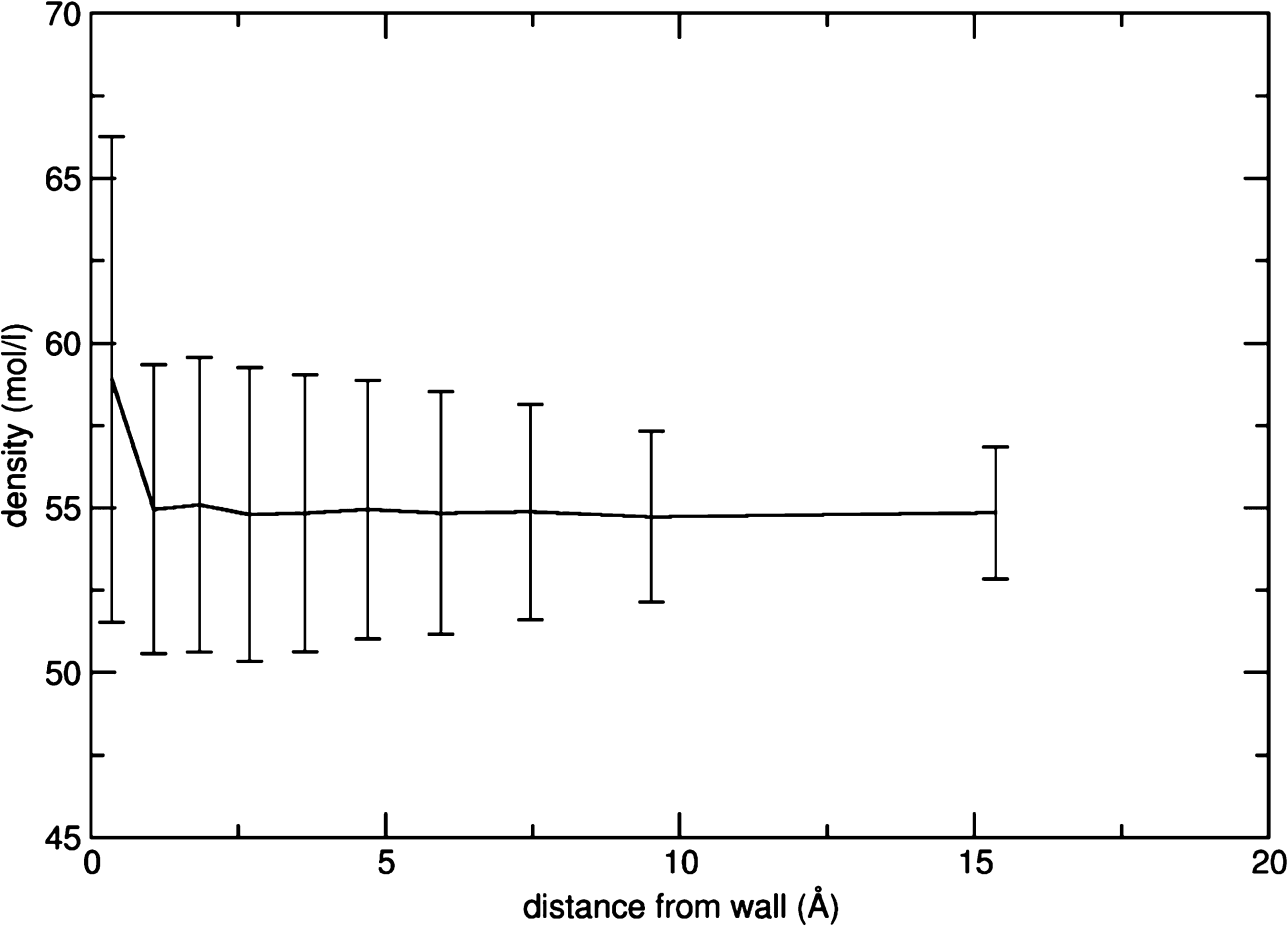
Average density and corresponding standard deviations in 10 concentric shells of constant volume for NPBC simulations of TIP3P-FB water.

At the end of the procedure, we ran an additional 10 ns simulation for each solvent in order to check the performance of the *U*_*vdW*_ term; Figure 6 clearly shows that the density fluctuations are small and are nearly constant within the whole simulation sphere.

### Application: TEMPO Radical in Different Solvents

#### NPBC Simulations

**Figure 7:**
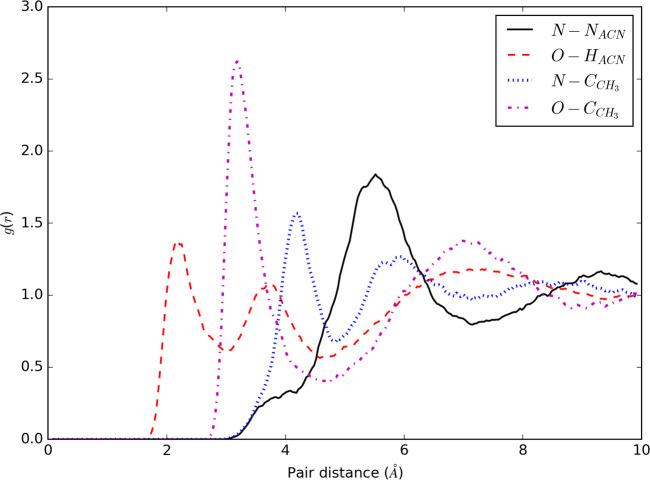
TEMPO–acetonitrile simulation: radial distribution functions.

The first simulation of TEMPO was carried out in acetonitrile, a polar non-hydrogen-bonding solvent. The resulting radial distribution functions for the oxygen and nitrogen distances from the methyl carbon (Figure 7) show two peaks with maxima located at 3.15 and 4.2 Å, respectively, which are indicative of an alignment of the methyl–cyanide dipole roughly antiparallel to the N–O one of TEMPO, which is confirmed by the position (2.2 Å) of the first peak maximum for the O–H_CH3CN_ pair.

The position of the N_TEMPO_–N_CH3CN_ first maximum at about 5.5 Å is indicative of little direct interaction above and below the average molecular plane of the solute. Anyhow, to include all possible contributions, we built the feature space using the dummy atom–methyl hydrogen and N–N first and second neighbor distances.

**Figure 8:**
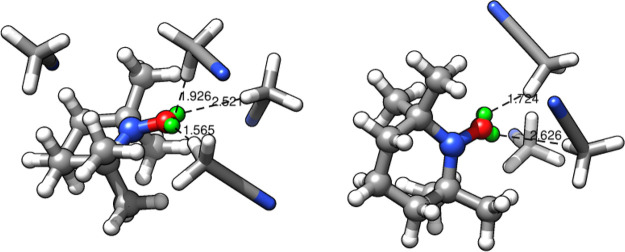
Cluster centroids of the TEMPO–CH_3_CN simulation. The TEMPO molecule (ball and stick) is shown with the solvent molecules (licorice) within 4 Å of the nitroxide group atoms (including the virtual sites). Some solute–solvent distances are indicated as well.

A subsequent PCA showed that using four coordinates yielded 91.1% of the total variance. The values of first and second neighbor distances are shown in Figure S1 of the Supporting Information. Two clusters of comparable size (389 and 552 members, respectively) were obtained by inspection of validation scores (Figure S2 of the Supporting Information), whose centroids are shown in Figure 8 and differ for the number of solvent molecules close to the nitroxide moiety.

**Figure 9:**
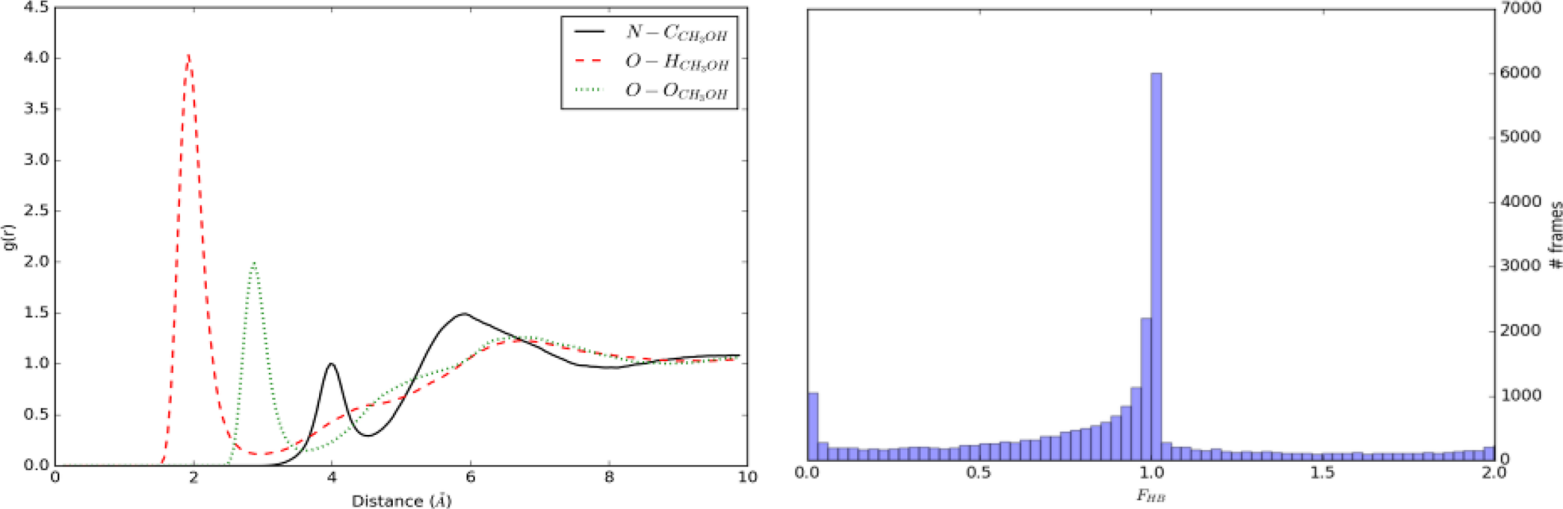
TEMPO–CH_3_OH simulation. Left panel: radial distribution functions for N–C, O–H, and O–O atom pairs. Right panel: histogram of the *F*_*HB*_ function.

The left panel in Figure 9 shows the radial distribution functions calculated from the TEMPO - CH_3_OH simulation. In this case, the *g*(*r*) shows sharp well-defined peaks and depletion zones for the atom involved in the hydrogen bond between methanol and TEMPO; the first maxima and minima are located at 1.93 and 2.88 Å, respectively. The *F*_*HB*_ histogram shows that one stable hydrogen bond is formed between TEMPO and methanol (⟨*F*_*HB*_⟩ = 0.896).

A PCA of the N–O, LP_1_–H, LP_2_–H, and O–O distances shows that 93.1% of the total variance is accounted for when using the first three components, which were then used for clustering (see Figure S3 in the Supporting Information). Inspection of the validation results (Figure S4 in the Supporting Information) indicates the presence of two clusters (three scores out of four) made up of 707 and 548 points, respectively. These were then passed to the GRASP step. The results of the PAM+GRASP procedure (see Figure S5 in the Supporting Information) show that the distributions sampled from the two clusters span the complete (irregular) shape of the feature space projected on its first two eigenvectors, whose corresponding eigenvalues are 0.78 and 0.11, respectively.

**Figure 10:**
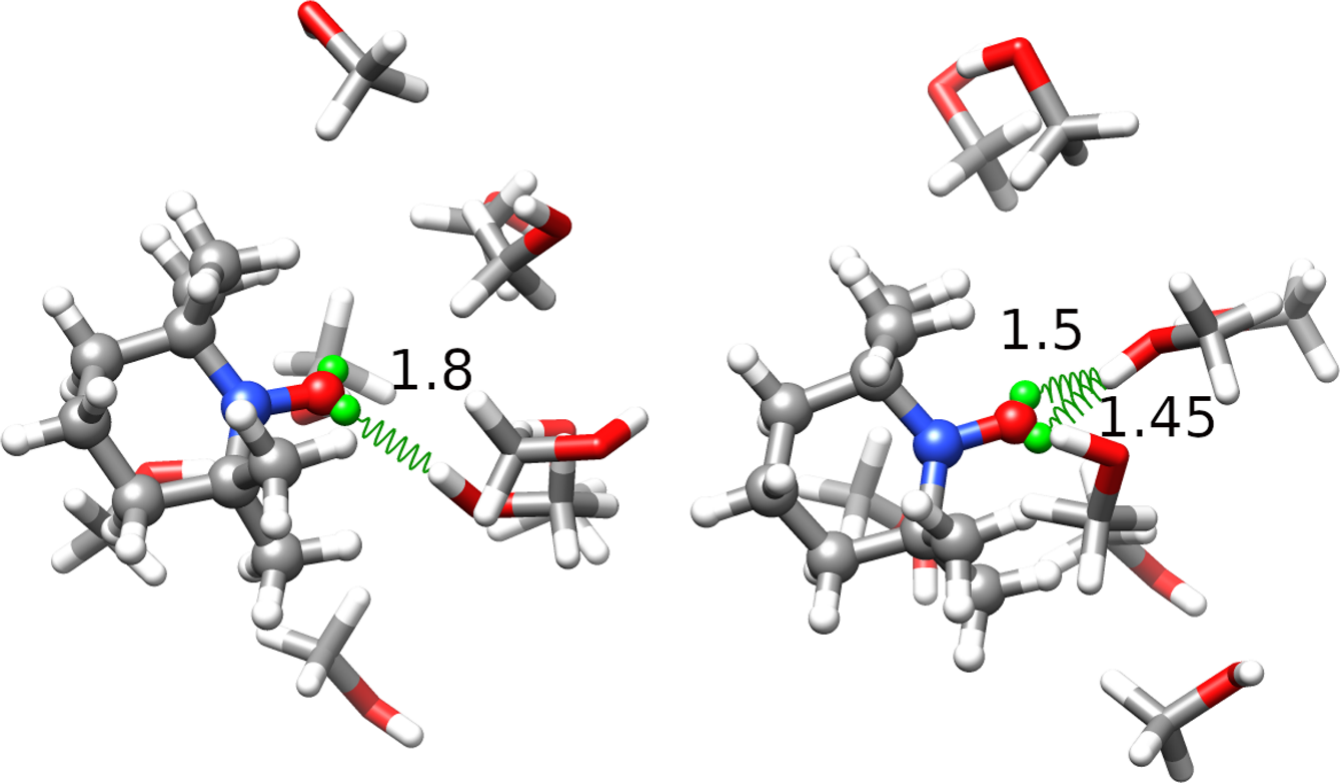
Cluster centroids of the TEMPO–CH_3_OH simulation. H-bonds are depicted as green springs.

Figure 10 shows a ball and stick representation of the two centroids including the solute and the solvent molecules within 4.0 Å of the atoms used to build the feature space, i.e., N, O, LP1, and LP2.

It is apparent that the left centroid, which corresponds to the most populated cluster, forms stable hydrogen bonds oriented along one of the virtual site-oxygen bonds. The average value of the *F*_*HB*_ function in this cluster is 0.89, very close to the value for the whole trajectory. In the right centroid, the nearest methanol molecule lies between the two solute virtual sites. In this cluster, ⟨*F*_*HB*_⟩ = 0.67, with this suggesting that the included frames account for solvent exchange events.

**Figure 11:**
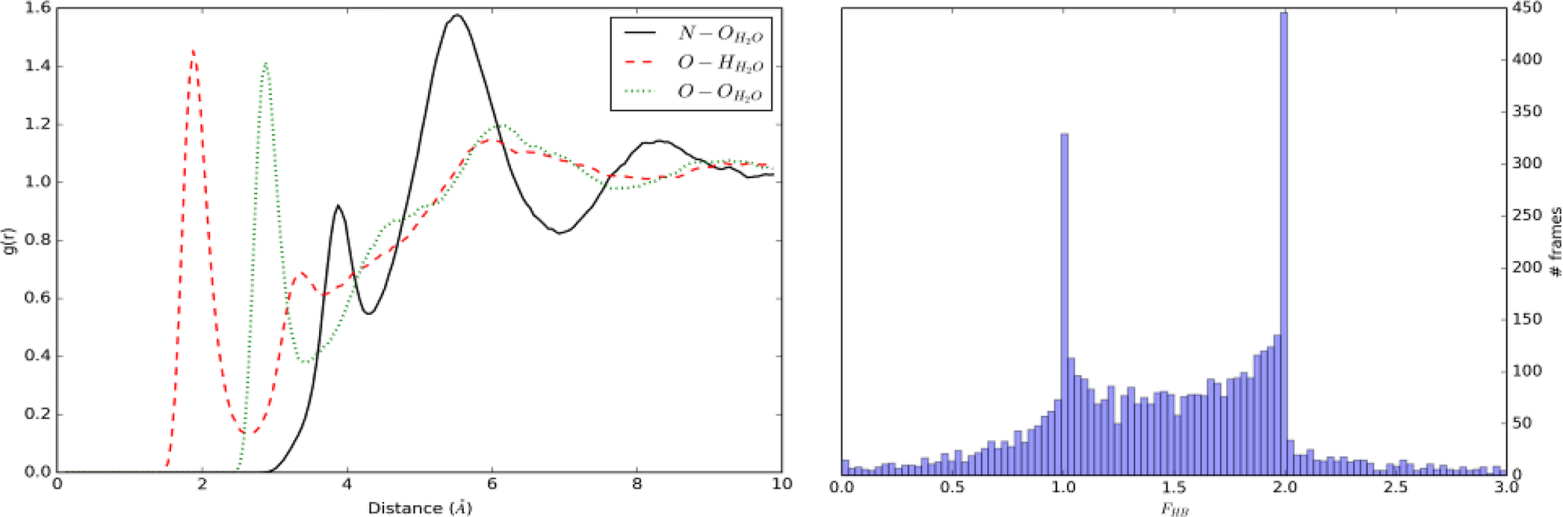
TEMPO–H_2_O simulation. Left panel: radial distribution functions for N–O, O–H, and O–O atom pairs. Right panel: histogram of the *F*_*HB*_ function.

The radial distribution functions obtained from the TEMPO–TIP3P-FB water trajectory are shown in Figure 11.

This trajectory shows stronger and more directional solute–solvent interactions, which are well represented by the positions of the *g*(*r*) first peaks, located at 3.88, 2.88, and 1.93 Å for N–O, O–H, and O–O, respectively, and the well-defined depletion zones separating the first and second coordination shell for O_TEMPO_–H_TIP 3p–FB_ and O_TEMPO_–O_TIP3P–FB_. The *F*_*HB*_ histogram shows a bimodal shape with peaks corresponding to one and two hydrogen bonds, respectively (⟨*F*_*HB*_⟩ = 1.467). It is noteworthy how the intermediate zone in the interval *F*_*HB*_ = [1, 2] is densely populated as well, indicating a continuous transition between the two most likely states.

The feature space employed for the frame selection was the same as that used for the TEMPO–methanol simulation, and the corresponding graphs are shown in Figure S6 of the Supporting Information. The filtered trajectory used to build the feature space included 941 points, and according to the corresponding PCA, five components are necessary to account for 90% of the total variance. The consensus of three scores out of four (see Figure S7 in the Supporting Information) led to the selection of three clusters containing 343, 425, and 173 points, respectively. Figure S8 in the Supporting Information is analogous to Figure S5, and the shape of the point distribution in the feature space is again well reproduced by GRASP. In this case, the eigenvalues of the first two principal components are 0.51 and 0.22, respectively.

**Figure 12:**
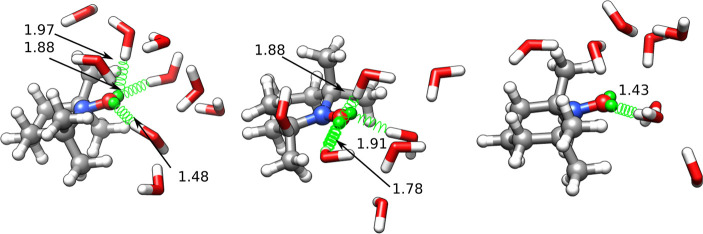
Cluster centroids of the TEMPO–H_2_O simulation. The same graphical conventions of Figure 10 have been used.

Figure 12 shows a ball and stick representation of the three centroids. In analogy with the methanol solution, the solvent molecules located within 4 Å of the atoms whose two nearest neighbor distances were used to build the feature space are shown together with TEMPO. The distances between water hydrogens and lone pairs of the nitroxide oxygen are indicated as well. It is apparent that the first two clusters involve water molecules close to both sides of the nitroxide moiety, at distances compatible with the formation of hydrogen bonds. The only exception is the molecule located at 1.48 Å in the leftmost panel, which is indicative of the same exchange phenomenon observed for methanol. Accordingly, the first two clusters show ⟨*F*_*HB*_⟩ values of 1.55 and 1.51, respectively. The third (and smallest) cluster shows a different structure with just one water molecule near a virtual site, and the average number of hydrogen bonds is reduced to 1.36.

The results of this subsection show that, as expected, the competition between solvent–solvent and solute–solvent interactions tunes the structure of the cybotactic region of TEMPO. In particular, one or two intermolecular hydrogen bonds can be formed by the lone pairs of the nitroxide oxygen, whose relative populations and interconversion rates depend on the nature of the solvent. Roughly speaking, water forms two stable hydrogen bonds, whereas methanol prefers a single hydrogen bond, possibly due to steric constraints by the methyl groups belonging to the solute and the solvent. The lack of solute–solvent hydrogen bonds in acetonitrile solution leads to a less rigid cybotactic region, although some order is apparent also in this case due to a preferential alignment of the solvent dipole moment roughly antiparallel to the NO one of TEMPO. Noted is that all these trends are in agreement with the available experimental evidence (see ref (45) and references therein).

### Solvatochromism of Nitrogen Isotropic Hyperfine Couplings

**Figure 13:**
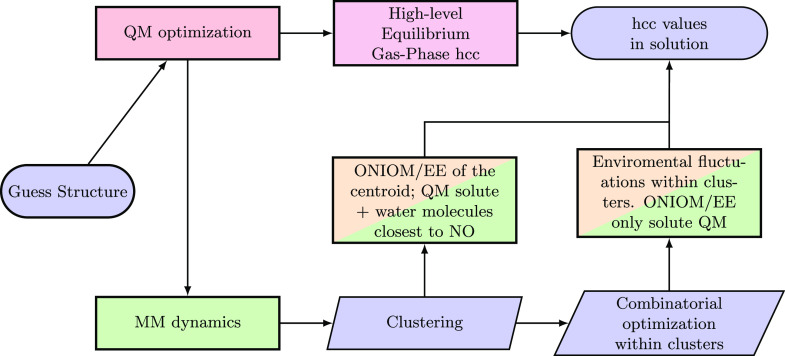
Flowchart of the computational strategy employed for the evaluation of TEMPO *A*_*N*_ in different solvents. Green boxes represent MM steps of the procedure, and orange boxes represent simulations typically performed with DFT and hybrid functionals, whereas the red ones are performed with double-hybrid functional. The purple box represents the step evaluated at the highest affordable level of theory (CC).

The computation of nitrogen isotropic hyperfine couplings (*A*_*N*_) for TEMPO in different solvents will be based on a validation of the QM model followed by an integrated strategy employing the results of the trajectory analyses described in the preceding section. The overall computational protocol is sketched in Figure 13, whose different steps will be analyzed in the following.

In order to define the most effective QC method to be employed in the present context, a small reference system (dimethyl nitroxide) was investigated by different hybrid functionals. We considered B3LYP,^46,47^ PBE0,^48^ and PW6B95,^49^ in conjunction with the double-ζ jul-cc-pVDZ^50^ and SNSD^51^ basis sets, and we also applied a last-generation double-hybrid functional rev-DSDPBEP86-D3^52^ (hereafter rDSD) with two triple-ζ basis sets, jun-cc-pVTZ^50^ and EPR(III).^53^ Reference geometries of both the pyramidal equilibrium structure and the planar transition state ruling the inversion have been obtained at the CCSD(T)-F12b(3C/FIX) level^54^ (hereafter referred to simply as CCSD(T)-F12) using the cc-pVTZ-F12 basis set,^55^ which is able to deliver results comparable with those obtainable by conventional CCSD(T) calculations employing much larger basis sets.^56^ Core–valence (CV) correlation effects, often mandatory for obtaining accurate geometrical parameters,^57^ were taken into account by adding to the results the difference between geometrical parameters optimized correlating all the electrons (AE) or freezing the core electrons (FC) using the cc-pCVDZ-F12 basis set.^58^ All the DFT computations were performed with the Gaussian suite of programs,^28^ employing, when needed, analytical first and second energy derivatives, whereas CCSD(T)-F12 computations were performed with the MOLPRO package,^59^ employing, when needed, numerical gradients.^60^

The results collected in Table 2 show small, but nonnegligible variations with respect to previous CCSD(T)-F12/cc-pVDZ-F12 results.^61^ Furthermore, both PW6B95 and rDSD results are quite accurate and represent significant improvements with respect to B3LYP and PBE0 values. This remark applies also to the inversion barrier, which is particularly important in connection with magnetic properties, especially in the case of the double-hybrid rDSD functional. It is noteworthy that the geometrical parameters obtained using basis sets purposely tailored for the computation of electron paramagnetic resonance (EPR) parameters (SNSD for hybrid functionals and EPR(III) for double-hybrid functionals) are close to those provided by the basis sets jul-cc-pVDZ and jun-cc-pVTZ for hybrid- and double-hybrid functionals, respectively.^62^ Consequently, only SNSD and EPR(III) basis sets will be employed in the following in conjunction with PW6B95 and rDSD functional, respectively.

**Table 2:** Geometrical Parameters of the Dimethyl Nitroxide Template Molecule at Different Levels of Theory^a^

		minimum				transition state			
level of theory	basis sets	*r*NO	*r*CN	CNC	CCNO	*r*NO	*r*CN	CNC	Δ*E* (cm^–1^)
B3LYP	SNSD	1.2818	1.4611	118.82	155.2	1.2808	1.4557	119.59	270.49
	julDZ	1.2818	1.4604	118.85	154.3	1.2817	1.4546	119.46	293.43
PBE0	SNSD	1.2683	1.4494	118.48	154.6	1.2688	1.4452	119.24	265.61
	julDZ	1.2700	1.4498	118.55	154.5	1.2702	1.4454	119.36	277.21
PW6B95D3	SNSD	1.2720	1.4506	118.75	155.9	1.2713	1.4458	119.63	264.70
	julDZ	1.2720	1.4495	118.82	154.6	1.2723	1.4452	119.67	271.85
rDSD-D3	junTZ	1.2754	1.4548	118.59	152.9	1.2765	1.4502	119.47	339.04
	EPR(III)	1.2743	1.4540	118.51	152.7	1.2752	1.4494	119.43	361.42
CCSD(T)-F12	DZ-F12	1.2773	1.4554	118.32	150.8	1.2774	1.4491	119.64	404.60
CCSD(T)-F12	TZ-F12	1.2762	1.4534	118.41	151.1	1.2764	1.4485	119.62	387.90
CCSD(T)-F12	TZ-F12+CV	1.2754	1.4517	118.41	151.2	1.2757	1.4469	119.60	371.45

a At all levels of theory the CCNO angle in the transitions state is 180.0°, and therefore, it was omitted from the table.

Next, the reliability of the computed isotropic hyperfine coupling of ^14^N (hereafter *A*_*N*_) has been checked using again dimethyl nitroxide and reference values obtained at the all-electron CCSD(T) level in conjunction with a large basis set including proper treatment of core electrons, namely aug-cc-pwCVQZ for s, p orbitals and aug-cc-pwCVTZ^63^ for the polarization functions. Test computations for H_2_NO showed that this basis set delivers nitrogen hyperfine couplings within 0.05 MHz from those obtained by the full aug-cc-pwCVQZ basis set. Furthermore, replacement of this basis set by the cheaper EPR-(III) counterpart for C and H atoms leads to very accurate results with a significant reduction of the computational cost. Note that the bare cc-pwCVTZ basis set delivers unsatisfactory results. All these computations have been performed with the CFOUR package.^64^

The results collected in Table 3 show that PBE0 and B3LYP functionals significantly underestimate the CCSD(T) reference values especially for pyramidal structures.The PW6B95 functional, which includes a larger amount of Hartree–Fock exchange, reduces the errors to 1.3 MHz for both planar and pyramidal structures, thus suggesting that environmental effects can be safely computed at this level. Along the same line, rDSD/EPR(III) results are in quantitative agreement with the reference values, with the difference (0.3 MHz) being within the experimental error bar for both planar and pyramidal structures. As far as the TEMPO radical is concerned, the *A*_*N*_ computed at the PW6B95/SNSD level is 41.0 MHz employing either PW6B95 or rDSD equilibrium structures, confirming that PW6B95 geometries can be confidently used for computing solvent effects. The difference of 1.3 MHz between the computed value of 41.4 MHz including a bulk solvent contribution estimated at the C-PCM level, and the experimental counterpart in cyclohexane solution (42.7 MHz^65^) is identical with its estimate obtained from the corresponding difference between *in vacuo* PW6B95 and rDSD results for TEMPO (1.0 MHz) plus the difference between rDSD and CCSD(T)/aug-cc-PwCV(Q,T)Z results for the pyramidal structure of dimethyl nitroxide (0.3 MHz). Taking into account the experimental error bar (0.3 MHz) and the uncertainties of the computational estimate, we will adopt in the following a correction of 1 MHz for all the PW6B95 results.

**Table 3:** Nitrogen Hyperfine Couplings in MHz for the Dimethyl Nitroxide Template Molecules Computed at Different Levels (Conversion Factors au MHz = 323.13 and au Gauss = 115.3)

level of theory	basis sets	minimum	TS
PBE0	SNSD	37.3	28.9
B3LYP	SNSD	41.7	28.5
PW6B95	SNSD	43.3	29.7
rDSD	EPR(III)	44.6	31.1
CCSD(T)	cc-pwCVTZ	40.6	28.2
CCSD(T)	aCV(T,Q)^a^	44.9	30.9
CCSD(T)	aCV(T,Q)/EPR(III)^a^	44.9	31.0

a aug-cc-pwCVQZ for s,p and aug-cc-pwCVTZ for d,f functions.

**Figure 14:**
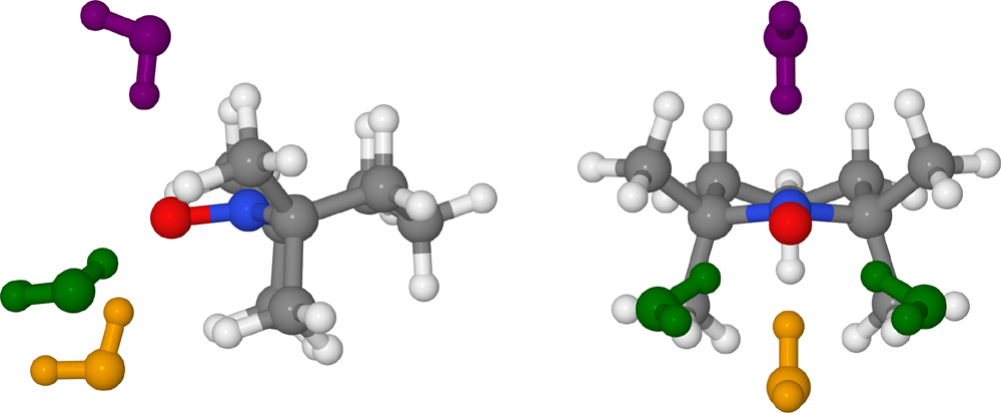
Representation of the relative positions of a water molecule with respect to the TEMPO NO group: **t**, orange; **p**, green; **o**, purple.

A first analysis of the role of solute–solvent interactions was performed by considering different clusters containing TEMPO and an increasing number of water molecules, according to the scheme shown in Figure 14

The results reported in Table 4 show that non negligible modifications of hyperfine coupling constants are induced by up to four water molecules near the NO moiety, with QM and MM representations of these water molecules leading to comparable results.

**Table 4:** Hyperfine Coupling Constants at the PW6B95/SNSD Level of Theory (Water Charges: O, −0.8; H, 0.4)

system	*a*_*N*_(QM)	*a*_*N*_(QM/MM)^a^	*a*_*N*_(QM+C-PCM)	Δ(QM)^b^	Δ(C-PCM)^c^
TEMPO	40.96	40.96	42.48	0.00	1.52
**o**	41.58	41.84 (0.26)	43.12	0.62	1.54
**t**	42.85	42.82 (−0.03)	44.76	1.90	1.91
**p**	42.37	42.09 (−0.28)	44.32	1.41	1.95
2·**p**	43.62	43.89 (0.27)	45.08	2.66	1.46
**o**+**t**	43.58	43.16 (0.42)	44.49	2.62	0.91
2·**p**+**o**	43.84	44.19 (0.35)	44.78	2.88	0.94
2·**p**+**t**	44.81	44.23 (−0.58)	46.21	3.85	1.40
**p**+**o**+**t**	44.17	44.54 (0.37)	45.08	3.21	0.91
2·**p**+**o**+**t**	45.68	45.11 (−0.57)	45.60	4.72	–0.08

a In parentheses is given the difference between QM and QM/MM. The mean absolute error and maximum error are 0.25 and 0.42 MHz, respectively.

b Differences between the different rows of column 2 and its first row (QM calculation for the bare TEMPO radical).

c Difference between the different rows of column 4 and the corresponding rows of column 2.

Next, a more systematic study of the difference between QM and MM (point charges) representations of solvent molecules has been performed for the centroids of the most populated clusters of TEMPO in acetonitrile, methanol, and water.

**Figure 15:**
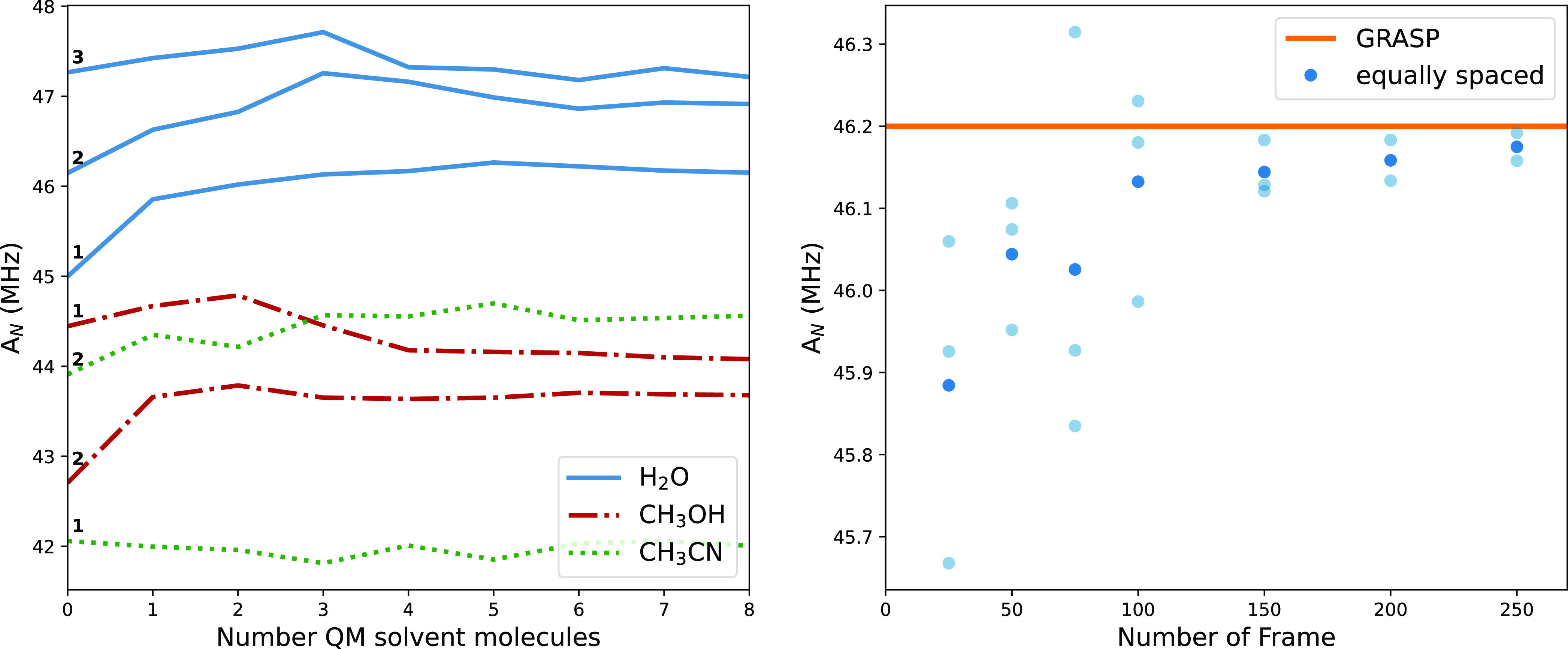
Convergence of TEMPO *A*_*N*_ as a function of the number of QM solvent molecules in different solvents (left panel, bold numbers identify the centroid) and of the number of equally spaced snapshots in aqueous solution with an MM (TIP3P-FB) representation of all the water molecules (right panel). In the latter case, the orange line corresponds to the value issued from the GRASP procedure (75 frames divided in three clusters) and the fading dots represent multiple evaluations starting from random points of the trajectory, whereas the bold dots are the mean values.

Figure 15 shows that converged results are always obtained using four solvent molecules described at the QM level. Furthermore, the right panel of the same figure confirms the efficiency of the clustering procedure, which allows one to reach the converged results, even for the most demanding solvent (i.e., water), with about one-third of the computational cost required when employing equally spaced snapshots.

Those findings suggest the following strategy based on the clustering of the trajectories. In the first step, the *A*_*N*_ of each cluster centroid is evaluated including the four solvent molecules closest to the NO moiety in the QM system embedded in the environment represented by the other MM water molecules. Next, fluctuations within each cluster are evaluated reducing the QM system to just the TEMPO radical, and the final value is increased by 1.0 MHz, as explained above. The results obtained for the different solvents are collected in Table 5. Note that CH_3_CN and CH_3_OH have similar dielectric constants, so that any bulk solvent model (here C-PCM^66^) gives close results and that saturation is already seen for dielectric constants above 35, leading to a very similar value also for water.

**Table 5:** Comparison between Computed and Experimental *A*_*N*_ for TEMPO Radical in Different Solvents

	CH_3_CN solution		CH_3_OH solution		H_2_O solution		
							
dielectric constant (ϵ)	35.688		32.613		78.3553		
C-PCM	42.4		42.4		42.5		
cluster number	1	2	1	2	1	2	3
cluster size	432	463	707	548	173	425	343
weight of the cluster	0.48	0.52	0.56	0.44	0.18	0.45	0.37
GRASP points	12	13	36	28	9	23	19
averaged a_*N*_(MM) in the cluster	42.8	43.1	43.8	43.5	46.7	46.0	46.3
a_*N*_(MM) at the centroid	42.1	43.9	44.4	42.7	47.3	46.1	45.0
a_*N*_(QM) at the centroid	42.0	44.6	44.2	43.8	47.3	47.1	46.2
weighted value	43.2		44.0		47.1		
best estimate^a^	44.2		45.0		48.1		
experiment	43.8^b^		45.4^b^		48.4^c^		

a weighted value + hybrid/double-hybrid difference.

b Reference (67).

c Reference (65).

On the contrary, the proposed computational procedure is able to disentangle the role of different interactions in the cybotactic region providing the correct trend of *A*_*N*_. The final results are indeed satisfactory also from a quantitative point of view when recalling that the estimated error of the experimental results is of the order of 0.3 MHz.

## Conclusions

In this paper, we have extended a general molecular dynamics tool capable of enforcing nonperiodic boundary conditions, by adding a very effective treatment (ddCOSMO) of reaction field effects from solvent bulk, and an isothermal isobaric ensemble. Furthermore, robust clustering procedures have been implemented in order to reduce the number of QM/MM computations to be performed to achieve well converged spectroscopic parameters. After a comprehensive test of the new tool for different conditions and optimization of the parameters for a new solvent, we have studied the solvatochromic shifts on the EPR spectra of a prototypical nitroxide radical (TEMPO). To this end, we have tuned the different computational parameters with reference to very accurate reference values for a small model system, showing that last-generation hybrid (PW6B95-D3) and double-hybrid (rev-DSDPBEP86-D3) functionals, in conjunction with purposely tailored basis sets (SNSD and EPR(III), respectively), deliver accurate results for both geometric and spectroscopic parameters. On these grounds, we have computed the nitrogen hyperfine coupling constant of a representative nitroxide radical (TEMPO) in different solvents. The good agreement of the results with the available experimental data confirms that we have at our disposal a robust and accurate tool for the study of spectroscopic parameters of medium-to-large size systems in condensed phases. Other fields of applications of the new tool can be also envisaged, including, for instance, fluxional atomic metal clusters or noble gas nanodroplets. In the first case, the recent development of synthetic techniques allowing for the production of monodisperse metal clusters composed by a few atoms^68^ paves the route toward bio-oriented applications.^69^ In the latter case, spectroscopic parameters (e.g., photoabsorption spectra) of molecules solvated in helium nanodroplets^70^ can be computed in the framework of a QM/MM model employing the recently developed renormalized He–He potentials to account for the fluid nature of the solvent.^71^ Work along these and related lines is ongoing in our laboratories.

## Supporting Information Available

Figures that describe the feature space and the results of clustering for the TEMPO–CH_3_CN, TEMPO–CH_3_OH, and TEMPO–H_2_O simulations (PDF)
