# Supplementary material for: Molecular Dynamics Simulations Enforcing Nonperiodic Boundary Conditions: New Developments and Application to the Solvent Shifts of Nitroxide Magnetic Parameters
Source: J Chem Theory Comput. 2022 Mar 8;18(4):2479–93. doi: 10.1021/acs.jctc.2c00046 (PMC9009096; doi:10.1021/acs.jctc.2c00046)
Supplement: Supplementary file 1 — ct2c00046_si_001.pdf [file ct2c00046_si_001.pdf]

Supporting Information: Molecular dynamics simulations enforcing non periodic boundary conditions: new developments and application to the solvent shifts of nitroxide magnetic parameters

Giordano Mancini,[*,†] Marco Fusè,[†] Filippo Lipparini,[‡] Michele Nottoli,[¶] Giovanni Scalmani,[§] and Vincenzo Barone[*,†]

†*Scuola Normale Superiore, Piazza dei Cavalieri 7, 56126 Pisa, Italy*

‡*Dipartimento di Chimica e Chimica Industriale, Università di Pisa, Via G. Moruzzi 13, 56124, Pisa, Italy*

¶*Dipartimento di Chimica e Chimica Industriale, Universitadi Pisa, Via G. Moruzzi 13, 56124, Pisa, Italy*

§*Gaussian, Inc., 340 Quinnipiac Street, Building 40, Wallingford, CT 06492, USA*

E-mail: giordano.mancini@sns.it; vincenzo.barone@sns.it

Clustering of TEMPO - solvent trajectories

In this section we report figures that describe the feature space and the results of clustering for the TEMPO-CH_3CN, TEMPO-CH_3OH, TEMPO-H_2O simulations. For each trajectory we show a graph which include the first and second neighbour distances between the nitroxide N atom and the nitroxide oxygen virtual sites as a function of time; the specific atoms used

for each solvent are reported in the main text. Additionally we show also the values of the validation score used (i. e. WSS, pSF, Dunn and Silhouette, see the Analysis of trajectories section in the main text) on the feature space for each trajectory.

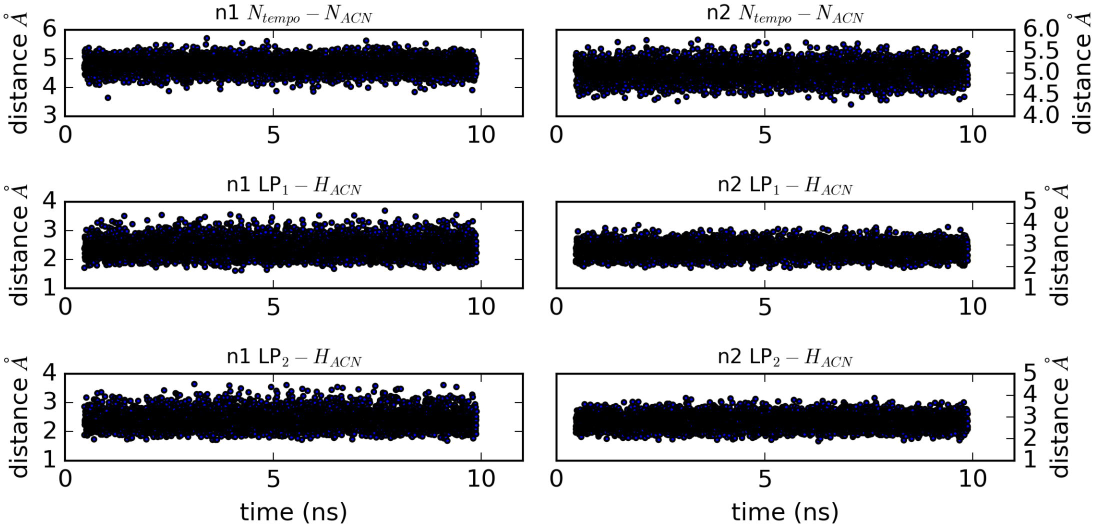

Figure S1: Tempo - CH_3CN simulation: first and second nearest neighbour solute - solvent distances as a function of time

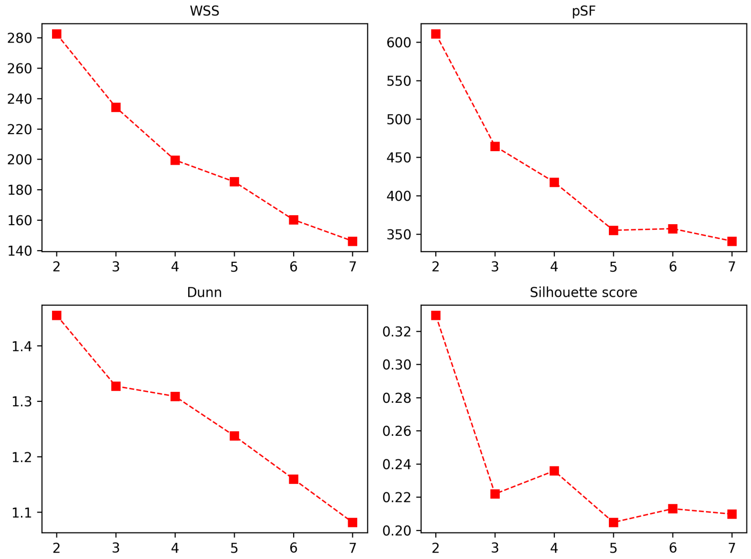

Figure S2: Within cluster sum of squares error (WSS), Calinski - Harabasz score (psf), Dunn Index (DI) and Silhouette coefficient (SI) computed for increasing k for the TEMPO - CH_3CN simulation

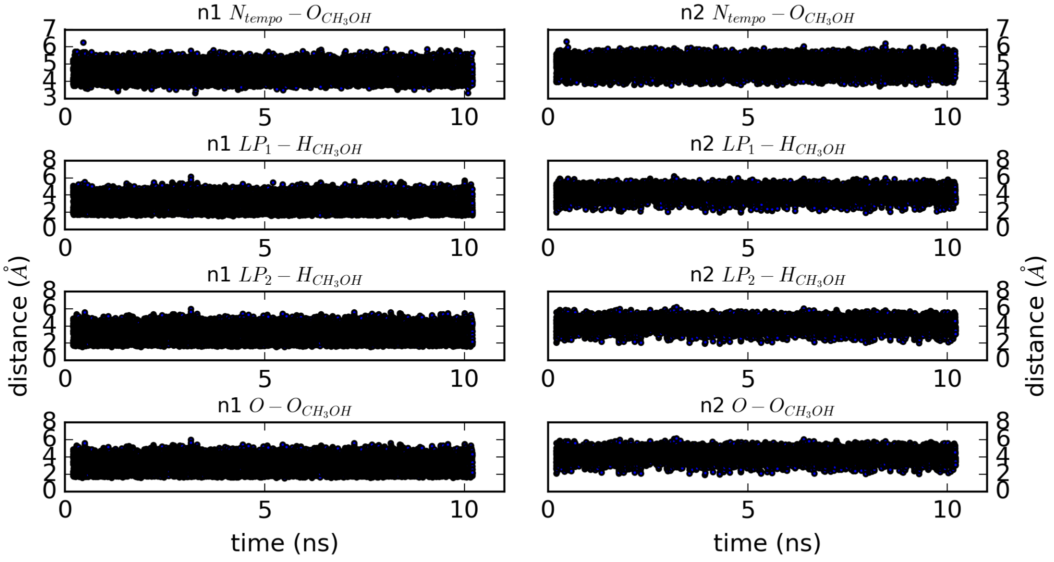

Figure S3: Tempo - CH_3OH simulation: first and second nearest neighbour solute - solvent distances as a function of time

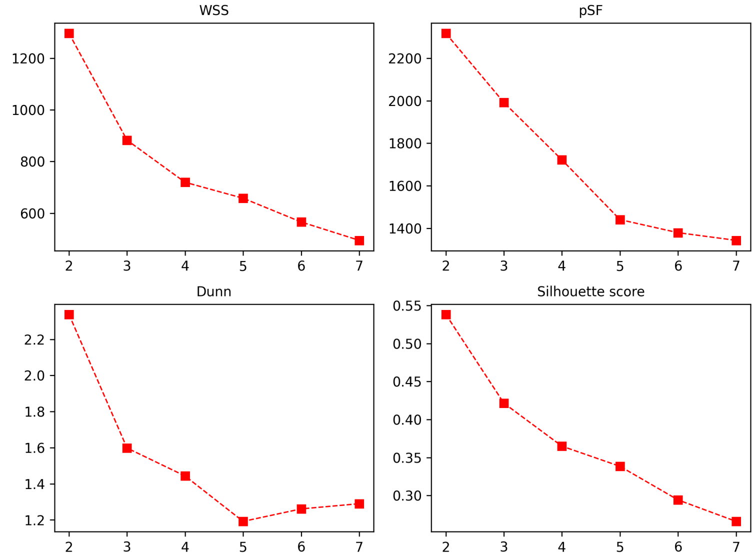

Figure S4: Within cluster sum of squares error (WSS), Calinski - Harabasz score (psf), Dunn Index (DI) and Silhouette coefficient (SI) computed for increasing k for the TEMPO - CH_3OH simulation

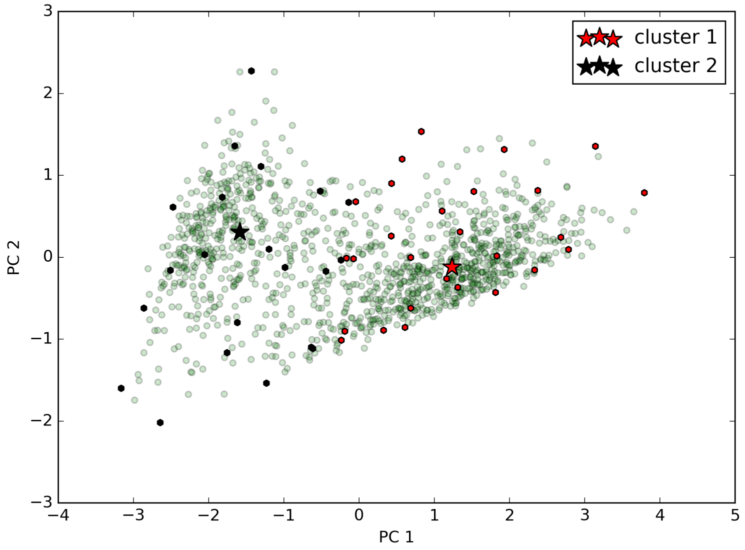

Figure S5: Clusters of TEMPO - CH_3OH simulations projected on the plane spanned by the first two principal components of the feature space. The green dots are complete data set (i. e. the trajectory sampled every 5 ps). The black and red stars represent the two centroids while the smaller points of the same color are the frames selected with GRASP within each cluster.

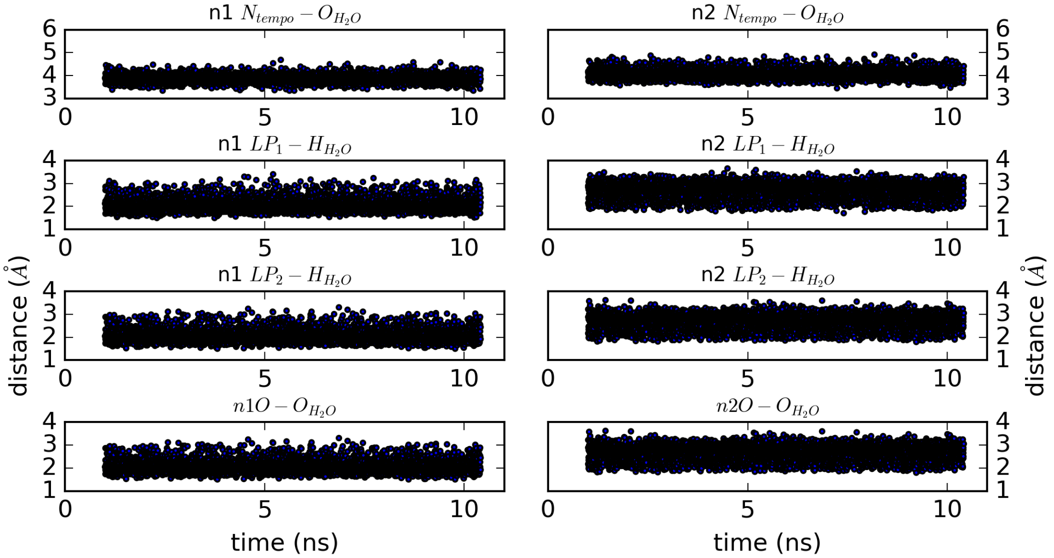

Figure S6: Tempo - TIP3P-FB water simulation: first and second nearest neighbour solute - solvent distances as a function of time

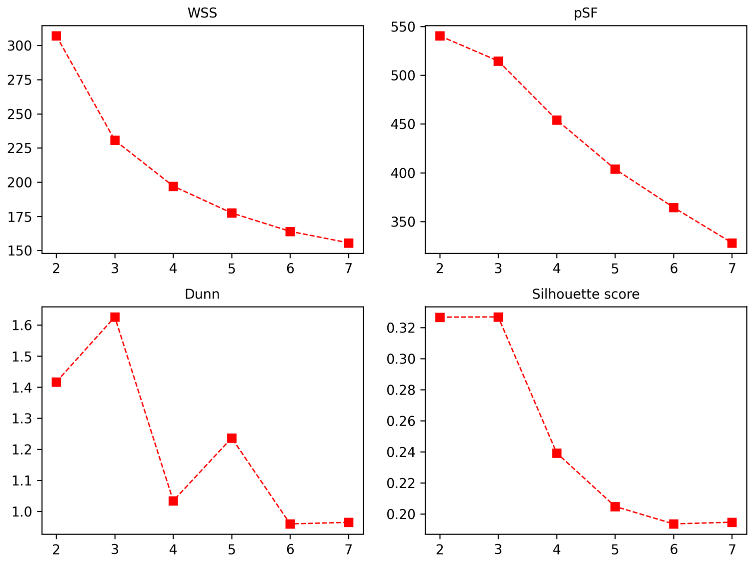

Figure S7: Within cluster sum of squares error (WSS), Calinski - Harabasz score (psf), Dunn Index (DI) and Silhouette coefficient (SI) computed for increasing k for the TEMPO - H_2O simulation

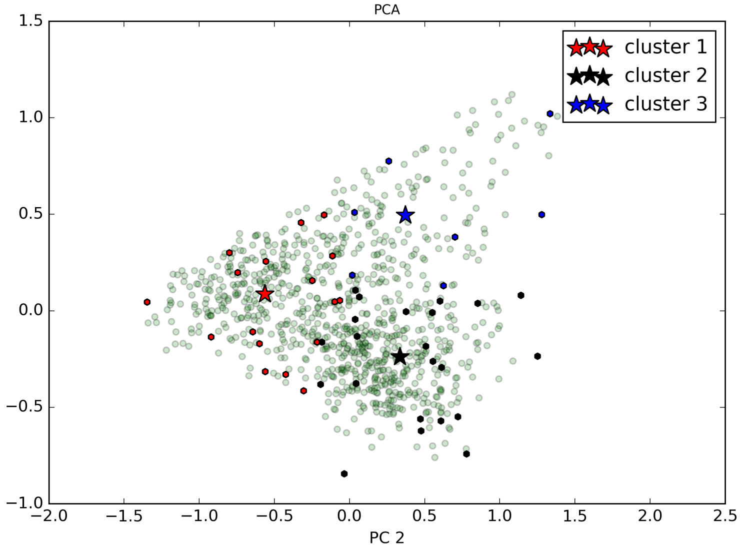

Figure S8: Clusters of TEMPO - H_2O simulations projected on the plane spanned by the first two principal components of the feature space. The green dots are complete data set (i. e. the trajectory sampled every 5 ps). The blue, black and red stars represent the three centroids while the smaller points of the same color are the frames selected with GRASP within each cluster.
